# Supplementary material for: Biosynthesis of Bacterial Nanocellulose from Low-Cost Cellulosic Feedstocks: Effect of Microbial Producer
Source: Int J Mol Sci. 2023 Sep 21;24(18):14401. doi: 10.3390/ijms241814401 (PMC10531556; doi:10.3390/ijms241814401)
Supplement: Supplementary file 1 [file ijms-24-14401-s001.zip › ijms-2560237-supplementary.pdf]

Supplementary Materials

Biosynthesis of Bacterial Nanocellulose from Low-Cost Cellulosic Feedstocks: Effect of Microbial Producer

Ekaterina A. Skiba [1,*], Nadezhda A. Shavyrkina [1], Maria A. Skiba [2], Galina F. Mironova [1] and Vera V. Budaeva [1,*]

[1] Laboratory of Bioconversion, Institute for Problems of Chemical and Energetic Technologies, Siberian Branch of the Russian Academy of Sciences (IPCET SB RAS), 659322 Biysk, Russia; 32nadina@mail.ru (N.A.S.); yur_galina@mail.ru (G.F.M.)

[2] Higher Chemical College of the Russian Academy of Sciences, Mendeleev University of Chemical Technology of Russia, 9, Miusskaya Square, 125047 Moscow, Russia; skibamaria21@gmail.com

* Correspondence: eas08988@mail.ru (E.A.S.); budaeva@ipcet.ru (V.V.B.)

Table of Contents

Table S1. Absorption band assignments 2

Chemical pretreatment of plant feedstocks 3

Table S2. Chemical compositions of *Miscanthus*, oat hulls and pulps 3

Enzymatic hydrolysis of pulps 4

Table S3. Enzymatic hydrolysis results for *Miscanthus*, oat hulls and pulps 5

Table S1. Absorption band assignments for functional groups of the BNC samples obtained from oat hulls and *Miscanthus* using individual Komagataeibacter xylinus strains and symbiotic culture.

Absorption band assignment*	Absorption band peak, cm^{-1}								
	Komagataeibacter xylinus B-12429			*Komagataeibacter xylinus* B-12431			*Medusomyces gisevii* Sa-12		
	MH	OHH	Control	MH	OHH	Control	MH	OHH	Control
ν OH groups involved in intermolecular and intramolecular H-bonding	3413	3435	3368	3411	3415	3379	3335	3432	3429
ν bonds in CH and CH2 groups	2916<br>2847	2916<br>2848	2916<br>2848	2914	2917<br>2850	2915	2919	2921	2920
δ HOH bonds due to tightly bound water present	1645	1639	1647	1640	1639	1641	1644	1643<br>1532	1654<br>1638
δ CH2 groups	1426<br>1372	1426<br>1371	1426<br>1369	1426<br>1369	1426<br>1369	1426<br>1369	1429	1431<br>1375	1430<br>1375
δ OH groups in CH2OH	1335<br>1315	1335<br>1315	1335	1335<br>1315	1336<br>1316	1335<br>1315	1316	–	–
δ CH2 groups in CH2OH	1278<br>1232	1278	1279	1279<br>1247<br>1233	1279<br>1248<br>1233	1279<br>1248<br>1233	1280	1281<br>1249	1282<br>1235
δ OH groups	-	-	-	-	1203	1203	1203	1204	–
ν C-O bonds (bands typical of polysaccharides due to present C-O-C acetyl bonds and C-O bonds in alcohols)	1161<br>1057	1162<br>1058	1160<br>1109<br>1057	1162<br>1111<br>1057<br>1032	1162<br>1110<br>1055<br>1030	1162<br>1110<br>1060<br>1033	1102<br>992	1102	1165<br>1060
β-1,4 linkages	894	894	850	896	895	896	899	899	899

1)* ν – stretching, δ – bending; MH – *Miscanthus* hydrolyzate; OHH – oat-hull hydrolyzate; 2) the controls were the Hestrin-Schramm medium for *Komagataeibacter xylinus* B-12429 and B-12431, and the synthetic medium supplemented with black tea extract for *Medusomyces gisevii Sa-12* ; 3) some of data on BNCs synthesized by *Medusomyces gisevii* Sa-12 were reported previously [25,26].

Chemical pretreatment of plant feedstocks [25]

The pretreatment was carried out under pilot production conditions at atmospheric pressure and 90–96 °C. A 250-L standard vessel was employed, and all the equipment and its piping are made of stainless steel.

The single feedstock loading was 10 kg. The pretreatment time was 14 h. The chopped *Miscanthus* or pristine oat hulls were pre-hydrolyzed with a 1% HNO_3 solution (AO KhimReaktiv, CAS No. 7697-32-7, Russia) at 96 °C for 1 h. Afterwards, the feedstocks were treated with a 4 % HNO_3 solution, washed with water, treated with a 4 % NaOH solution (OAO Kaustik, CAS No.1310-73-2, Russia), and then washed with water until natural wash waters. The resultant pulps were squeezed in a vacuum filter and used as substrates in the wet state for enzymatic hydrolysis.

Table S2 lists the chemical compositions of the feedstocks and the resultant pulps.

Table S2. Chemical compositions of *Miscanthus*, oat hulls and pulps [69].

**Measures**	***Miscanthus***	***Miscanthus* pulp**	**Oat hulls**	**Oat-hull pulp**
α-Cellulose, %	50.3±0.2	95.8±0.3	44.9±0.2	94.2±0.2
Pentosans, %	22.7±0.1	2.5±0.1	31.4±0.1	3.8±0.1
Acid-insoluble lignin, %	21.4±0.1	1.5±0.1	18.8±0.1	0.8±0.1
Ash content, %	5.6±0.05	0.2±0.05	4.9±0.05	1.2±0.05
Total hydrolyzables, %	72.3±0.3	98.3±0.3	76.3±0.3	97.8±0.3

Enzymatic hydrolysis of pulps [69]

The enzymatic hydrolysis of the resultant substrates was run in an 11-L fermentor in an aqueous medium at 47±2°C for 72 h by using the enzymes CelloLux-A (Sibbiopharm Ltd, Russia) and BrewZyme BGX (Tarchomin Pharmaceutical Works Polfa S.A., Poland) standardized against cellulase, xylanase and β-glucanase in compliance with their analytical datasheets. The enzyme cocktail was injected as follows: CelloLux-A 40 FPU/g solid and BrewZyme BGX 15 FPU/g solid. FPU was determined by the international procedure The pH was maintained at 4.7±0.2 with ammonium hydroxide (OOO KhimPostavka, CAS No. 1336-21-6, Russia) and orthophosphoric acid (OOO KhimSpetsializatsia, CAS No. 7664-38-2, Russia). The initial solid loading was 30 g/L.

The yield of reducing sugars (RS) was calculated by Eq. (1):

$$\eta_{RS} = \frac{C_F \cdot V_H}{m_S} \cdot 0.9 \cdot 100 \qquad (1)$$

where η_{RS} is the RS yield on a solid weight basis (%);

C_F is the final RS concentration in the hydrolyzate (g/L);

V_H is the enzymatic hydrolyzate volume (L);

0.90 is the coefficient attributed to the water molecule addition to anhydroglucose residues of the corresponding monomer units as a result of enzymatic hydrolysis;

m_S is the weight of the substrate for fermentation (g).

Table S3 summarizes enzymatic hydrolysis results for the feedstocks and pulps.

Table S3. Enzymatic hydrolysis results for *Miscanthus*, oat hulls and pulps.

Measures	*Miscanthus*	*Miscanthus* pulp	Oat hulls	Oat-hull pulp
RS concentration, g/L	4.1±0.1	26.7±0.2	4.5±0.1	24.5±0.2
RS yield on a substrate weight basis, %	11.1±0.3	72.1±0.3	15.8±0.3	73.5±0.3
Glucose concentration, g/L	4.0±0.1	26.5±0.2	4.4±0.1	22.3±0.2
Xylose concentration, g/L	0.10±0.05	0.20±0.05	0.10±0.05	2.20±0.05
Once standardized against reducing sugars				
RS concentration, g/L	–	20.0±0.2	–	20.0±0.2
Xylose concentration, g/L	–	0.15±0.05	–	1.80±0.05
